# Single-cell motile behaviour of ${Trypanosoma\, brucei}$ in thin-layered fluid collectives

**DOI:** 10.1140/epje/s10189-021-00052-7

**Published:** 2021-03-23

**Authors:** Timothy Krüger, Katharina Maus, Verena Kreß, Elisabeth Meyer-Natus, Markus Engstler

**Affiliations:** Lehrstuhl für Zell- und Entwicklungsbiologie, Biozentrum, Julius-Maximilians-Universität, Am Hubland, 97074 Würzburg, Germany

## Abstract

**Abstract:**

We describe a system for the analysis of an important unicellular eukaryotic flagellate in a confining and crowded environment. The parasite *Trypanosoma brucei* is arguably one of the most versatile microswimmers known. It has unique properties as a single microswimmer and shows remarkable adaptations (not only in motility, but prominently so), to its environment during a complex developmental cycle involving two different hosts. Specific life cycle stages show fascinating collective behaviour, as millions of cells can be forced to move together in extreme confinement. Our goal is to examine such motile behaviour directly in the context of the relevant environments. Therefore, for the first time, we analyse the motility behaviour of trypanosomes directly in a widely used assay, which aims to evaluate the parasites behaviour in collectives, in response to as yet unknown parameters. In a step towards understanding whether, or what type of, swarming behaviour of trypanosomes exists, we customised the assay for quantitative tracking analysis of motile behaviour on the single-cell level. We show that the migration speed of cell groups does not directly depend on single-cell velocity and that the system remains to be simplified further, before hypotheses about collective motility can be advanced.

**Graphic abstract:**

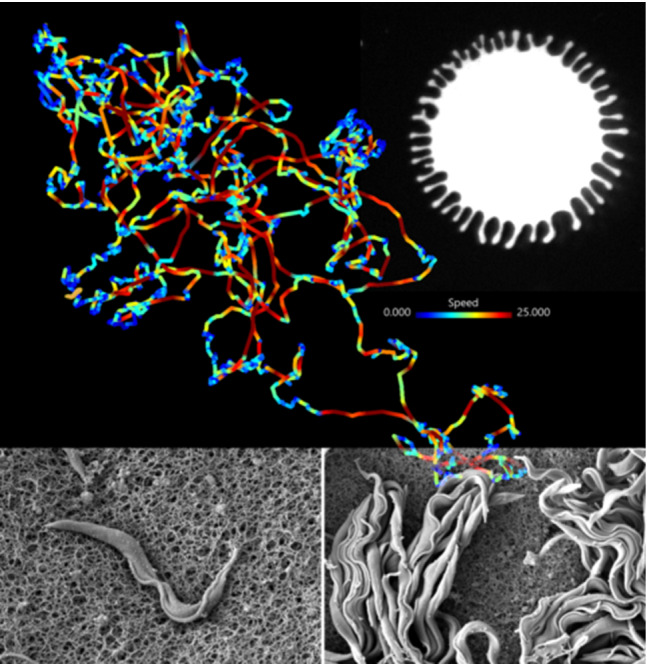

**Supplementary Information:**

The online version supplementary material available at 10.1140/epje/s10189-021-00052-7.

## Introduction

The unicellular parasite *Trypanosoma brucei* morphs through a series of developmental stages during its life cycle in the tsetse fly vector and its mammalian hosts [1]. The uniflagellated, fusiform morphotypes have characteristic lengths and motility behaviours [2, 3]. The procyclic form is the first type to develop after the cells are taken up by the fly during bloodmeals. Its natural habitats are accordingly the microenvironments of the tsetse fly’s midgut, where it swims solitarily and in groups, which can grow into huge swarms of aligned and synchronising cells under confinement [3]. The procyclic culture form (PCF) is also well known as a vivid swimmer in culture, where it reaches relatively high densities but does not give rise to swarms. In all cases, the cells show Lévy walk-type swimming trajectories, with long stretches of directional forward movement at speeds of up to 20–40 $\upmu \hbox {m/s}$, interrupted by short stops and direction changes [3, 4].

Effective growth of the fly parasite *Trypanosoma brucei* in colonies was first described by [5] on semi-solid agarose gel surfaces. Later, an eye-catching motile behaviour of such colonies was documented, where hundreds of thousands of parasites spread as a collective on semi-solid gel surfaces in radial projections. The spreading was shown to be influenced by neighbouring colonies and therefore called social motility [6]. Since then, social motility assays have been used to characterise various motility and signalling defective mutants and the behaviour has been hypothesised to reflect the parasites behaviour in the host, the tsetse [7–10]. The motility of fly stage trypanosomes in large swarms was documented on the single-cell level by [3].

Social motility assays are akin to bacterial swarming experiments, in which a large colony of cells is observed to grow and move on the surface of a nutrient retaining hydrogel, typically an agar or agarose gel. The expansion of the colony is observed, and motile cells are shown to collectively move in intricate patterns, a behaviour called swarming or flocking [11, 12]. The analysis of such swarming systems has received much attention in the last few decades, but the magnitude of underlying mechanisms has only begun to be tackled [13–15]. Most studies have been performed with prokaryotes, as collectively swimming, single-cell eukaryotes are rare or do not find themselves amongst the model systems. *Trypanosoma brucei* is a unique model organism and a well-analysed uniflagellate microswimmer [2, 16–20]. As such, the parasite is ideally suited for the analysis of a variety of single cell and collective motility behaviours.

In the trypanosome collective motility assays used so far, cell numbers in the range of $10^{5}$-$10^{6}$ cells are spotted on the surface of an 0.4 % agarose gel. The medium in cell culture drop evaporates and is absorbed by the gel, leaving the cells highly concentrated in a disc a few millimetres in diameter. The parasites are thus trapped in a layer between the air and the surface of the hydrogel. In the volume of this fluid layer, they swim in a tightly packed mass. The Petri dish in which the gel is cast, is sealed and incubated at optimal culture conditions for the trypanosomes. Depending on the cell type and concentration, the cell layer will begin to expand radially, thereby producing projections or “fingers” that emanate from the circular rim of the original spotted culture drop. The hallmark migratory phase of trypanosome social motility can continue for days on the gel surface, during which the projections reach the periphery of the Petri dish or the projections of neighbouring colonies migrating in the opposite direction. These opposing fingers tend to divert each other from the more or less unidirectional (often curved) radial path, producing bent trajectories. This interaction has been interpreted as a form of inhibitory communication, which gave rise to the naming of social motility.

As this behaviour was hypothesised to play a role in the migration and development of the parasites in their fly vector, in previous work, we had infected flies with mixed populations of fluorescent and non-fluorescent trypanosomes, in order to track single fluorescent cells in the tightly packed swarms of parasites in the organs of the tsetse host [3]. We aimed to use the same strategy in the better controllable and accessible setting of the surface trapped trypanosome colonies, in order to analyse the motility of single parasites in collective motility assays.

Not only the single-cell behaviour in the original assay was unknown, but also the physiochemical basis of the cell culture drop spreading on the seemingly simple agarose gel surface. On the one hand, the presence or absence of several substances in the nutritional medium, e.g. glucose, glycerol, lipids from serum, will change fluid properties such as osmolarity, viscosity or fluidity of the porous hydrogel. These in turn will influence collective behaviour patterns of the cells spreading on its surface. On the other hand, the conditions used for culture form trypanosomes do not correspond to the conditions in their natural environment in the fly gut. Instead of glucose, which is the main nutritional source for culture and bloodstream forms, the fly-provided amino acids, mainly proline, are metabolised by the procyclic form parasites [21, 22].

For these reasons, we describe here a downscaled migration assay, that was established empirically by the adjustment of several parameters, in order to reduce the complexity of the visco-dynamic system, to approximate more natural environmental conditions, and to simultaneously track single-cell behaviour and macroscopic development of cell colonies in a quantifiable manner.

## Materials and methods

### Cell lines

AnTat 1.1: *Trypanosoma brucei brucei* strain EATRO 1125, serodeme AnTat1.1 [23].

AnTat 1.1 dtTomato: Bloodstream form (BSF) AnTat 1.1 was transfected with a pTSARib-dtTomato plasmid expressing puromycin resistance [24]. BSF was differentiated into procyclic culture forms (PCF) according to [25].

### Cell culture

Both cell lines were kept in SDM79 supplemented with 10 % heat inactivated fetal bovine serum (FBS), 7,5 mg/l hemin and 20 mM glycerol (SDMG). They were additionally cultured in SDM79 without added glucose, hemin and glycerol, supplemented with 10 % heat inactivated FBS. In $\hbox {SDM}^{\text {-}\mathrm{glc}}$, the glucose concentration was reduced from 6 mM in SDM79 to 0,5 mM (D-Glucose from FBS). Cultures were diluted to a concentration of $10^{6}$ cells/ml every two or three days.

Population doubling times (PDT):

AnTat 1.1 (wild type) in SDMG: 20,3 h ± 3,5 h; in $\hbox {SDM}^{\text {-}\mathrm{glc}}$: 22,5 h ± 3,7 h.

AnTat 1.1 dtTomato in SDMG: 20,6 h ± 3,3 h; in $\hbox {SDM}^{\text {-}\mathrm{glc}}$: 22,4 h ± 4,9 h.

### Agarose gels

4 % agarose (Promega, V3121) was dissolved and brought to the boil in $\hbox {dH}_{2}\hbox {O}$ under stirring and cooled to $50\,^{\circ }\hbox {C}$. Agarose was added to prewarmed medium ($50\,^{\circ }\hbox {C}$) to a final concentration of 0,4 %. The solution was quenched at $50\,^{\circ }\hbox {C}$ for 30 min and then pipetted into Petri dishes. (10 ml in 100 mm Ø dishes, 4 ml/60 mm Ø, 2ml/35 mm Ø). The agarose medium was left to gel for 30 min to 1 h in a biological safety cabinet with lids covering the surfaces, but leaving sufficient space not to restrict air flow between lid and gel surface. The gels were used immediately for motility assays.

### Motility assays

AnTat 1.1 and AnTat 1.1 dtTomato cells were grown in 10ml SDMG or $\hbox {SDM}^{\text {-}\mathrm{glc}}$ to concentrations of $8^{*}10^{6}$–$1,5^{*}10^{7}$ cells/ml (equal density for each experiment). Directly before gels were ready to use, $25\,\upmu \hbox {l}$ of dtTomato cells was added to the wild-type cells and 8 ml of each culture was centrifuged at $1400^{*}\hbox {g}$ and $27\,^{\circ }\hbox {C}$ for 8 min. The supernatant was aspirated, leaving a volume of ${\sim }\,100\,\upmu \hbox {l}$, in which the cells were carefully but thoroughly resuspended. Of this concentrated cell suspension, ($8^{*}10^{8}$ cells/ml, assuming $1^{*}10^{7}$ cells/ml in cell culture), $5\,\upmu \hbox {l}$ (for the 100 mm Ø gels) or $3\,\upmu \hbox {l}$ (for the smaller gels) were pipetted onto the surface of the freshly prepared gel surface. Care was taken to not disturb the gel surface and apply the volume in one single drop that spread in an even circle. The procedure was controlled with a stereo microscope. The dishes were immediately closed, sealed with Parafilm and incubated at $27\,^{\circ }\hbox {C}$ and 5 % $\hbox {CO}_{2}$.

### Fluorescence imaging and microscopy

Gels with AnTat1.1 dtTomato cells were visualised by the fluorescence documentation system iBright CL1000 (Invitrogen). Exposure time was 100 ms. Images were combined into TIF-stacks with Fiji, and the colonies were aligned using the Plugin “Linear Stack Alignment with SIFT”. The projection expansion speeds were measured by manually drawing the tangents normal to the longitudinal centre line of each projection tip in successive frames and measuring the distances in Fiji.

Microscopy was performed with a fluorescence stereomicroscope (MZ16 FA, Leica), equipped with a 5x zoom objective and a CCD camera (pco.1600, pco), and with an inverted fluorescence microscope (DMI6000B, Leica), equipped with an automated stage (Pecon) and an incubation chamber (Leica). Sixty-millimetre and 35-mm gel dishes were fixed in the appropriate dish holders and incubated at $27\,^{\circ }\hbox {C}$ and 5 % $\hbox {CO}_{2}$. Single projection tips were recorded at various time points by using a 20x, NA 0,4 phase contrast objective, in combined fluorescence and phase contrast mode (FLUO-PH, LASX software, 5 ms exposure time, minimal transmitted light). Recordings were 15 min each with a temporal resolution of 250 ms (4 fps).

### Tracking

Tracking of the ~0,3 % fluorescent AnTat 1.1 dtTomato cells in the wild-type cell projection tips was performed with Imaris (9.5.1, Oxford Instruments). The cells were classified using the surface module and tracked using the “Autoregression Motion” tracking algorithm. Tracks were controlled for specificity and colour coded to visualise distribution of time and speed data directly over time periods and regions adjustable at will. Data plots were generated automatically using the Vantage module. Instantaneous speed data were plotted directly. Track data were edited manually to combine track segments that the algorithm detected with interruptions, due to crossing events of two or more cells, or high background signal, *i.e*. at the borders of the dish.

### Scanning electron microscopy

Agarose gels were produced leaving a space between the Petri dish side wall and the agarose pad. This space was filled gradually with Sörensen’s fixation buffer (6,25 % glutaraldehyde in 50 mM phosphate buffer, pH 7,4), so as to disturb the cell layer on the gel surface as little as possible. The gel with the trypanosome layer was fixed for 30 min at room temperature or overnight at $4\,^{\circ }\hbox {C}$. The samples were washed thrice for 1 h at $4\,^{\circ }\hbox {C}$ with Sörensen’s buffer, always taking care to keep the fixative level just below the cell layer. Serial dehydration was performed in acetone, again keeping the fluid underneath the cell layer. After dehydration, the cell layer was submerged in 100% acetone. The specimens were critical point dried and coated with gold-palladium. Scanning electron microscopy was performed with a JSM-7500F field emission scanning electron microscope (JEOL).

Figure panels were arranged and annotated using the Fiji plugin ScientiFig (v3.2) [26].

## Result

**Fig. 1:**
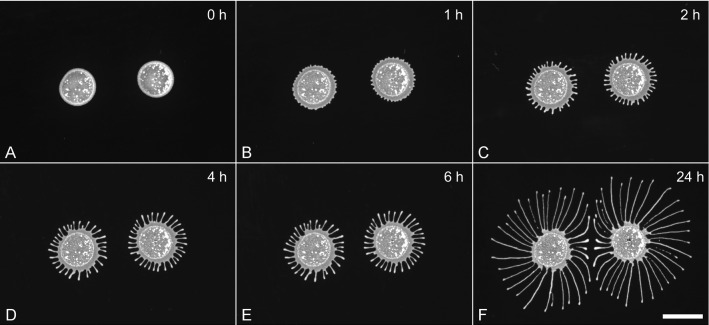
Social motility assay with two colonies of AnTat-dtTomato cells. Two $5\,\upmu \hbox {l}$ drops, containing $5*10^6$ cells each, were pipetted approximately 1 cm apart on a 0,4 % SDM79-agarose gel with a diameter of 10 cm. The gel was incubated at $27\,^{\circ }\hbox {C}$, $5\, \% \,\hbox {CO}_{2}$. The migration of projections was recorded hourly by fluorescence imaging. Scale bar 10 mm

We first tested the ability of a wild-type cell line expressing a fluorescent marker (tdTomato) to perform social motility. Average cell growth rates of AnTat 1.1 dtTomato were very similar to wild-type population growth rates (Materials and methods). In the standard assay, $5\,\upmu \hbox {l}$ drops of an exponentially growing ($8^{*}10^{6}$- $1,5^{*}10^{7}$ cells/ml) and freshly concentrated cell culture (to ${\sim }\,10^{6}\hbox {cells}/\,\upmu \hbox {l}$) were pipetted on a 0,4 % agarose gel, sealed with parafilm and incubated for 24 h at $27\,^{\circ }\hbox {C}$ and 5% $\hbox {CO}_{2}$. The colonies spread radially and started to exhibit instabilities around the migrating rim as soon as 1 or 2 h later (Fig. 1a–c). Numerous small protrusions were extended from the entire periphery in the following, producing continuously migrating fingers, radiating uniformly in all directions (Fig. 1c–e). The border of the troughs mostly remained in the same position, receding slightly at later time points. At the centre of the distance between two neighbouring colonies, the finger’s migration was inhibited or deflected by opposing cell projections, keeping them separated at about a finger’s width (Fig. 1f). This behaviour paralleled the collective motility behaviour shown in [6], albeit the onset was earlier and the speed of migration was higher, probably due to the higher starting concentration used here. A dependency of the migration time points on cell concentration had been shown by [7]. The migration speed of individual projections measured by hourly fluorescence imaging, as shown in Fig. 1, fluctuated in the range of 0–1 mm/h (mean $0,41\hbox { mm/h}\pm 0, 14\hbox { mm/h}$). The migration speed varied, both between different projection tips at different time points and time points of each individual projection (Fig. S1). The migration speeds were thus in the reported region “of a few microns per minute” and higher [6].

**Fig. 2:**
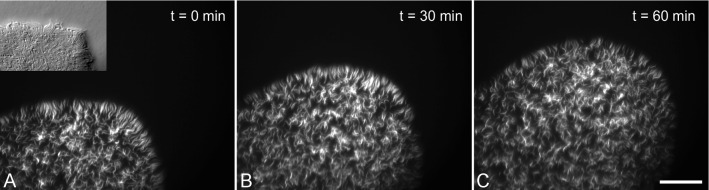
Trypanosomes confined to a dense monolayer in a radially expanding projection tip. 5 % AnTat 1.1 dtTomato cells were added to non-fluorescent cells before the experiment, which was performed as described in Fig. 1. The projection tip was recorded by fluorescence stereo microscopy for 1 min every 30 min. Projection migration speed was $6,5\,\upmu \hbox {m/min}$ from **a** to **b** and $5,5\,\upmu \hbox {m/min}$ from **b** to **c**. The inset shows a modulation contrast image of a similar experiment. The cells are oriented with the anterior tip of the flagellum mostly heading outwards and with varying degrees of lateral alignment. Scale bar: $100\,\upmu \hbox {m}$

At higher magnification, the trypanosomes were visible as a tightly packed mass of highly motile cells (Fig. 2a, inset). This had been observed before, but behaviour on the single-cell level had not been analysed further [6, 27]. The cells were free to swim in the central region of the projections. They were stopped by the fluid border, facing outwards with the anterior flagella tip, and reversed periodically from that position back to the more fluid centre (Video S1). The cells showed considerable alignment during the visit to the projection rim, but the alignment was temporary and cells were mostly able to wriggle in the thin fluid region surrounding the bulk (Fig. 2 and Video S1). Importantly, they were in the focus range across the entire field of view, meaning they were swimming in one tightly packed layer on the gel surface. We tested mixing fluorescent and non-fluorescent cells in different ratios, in order to visualise single-cell behaviour and evaluate the feasibility of tracking experiments (Fig. 2 and Video S1, dilution 1:20).

We then attempted to visualise this layer of tightly packed cells by electron microscopy. As the topology of the trypanosome-gel interface was hitherto unknown, but could be decisive for the cell’s motile behaviour, we wanted to visualise the status of the cells and verify the nature of the underlying gel surface. The polymer mesh of agarose gels has been shown to have an average pore size in the range of 300–600 nm for an agarose concentration of 0,4 % [28] and therefore retains the trypanosomes on the surface, as the cells width is around $2-3\,\upmu \hbox {m}$. As the measurements of pore sizes become more variable in low percentage gels, depending on the type of measurement used, and are likely dependent on the medium, we reckon the nanoscopic analysis to be important for characterisation of surface swarming behaviour.

**Fig. 3:**
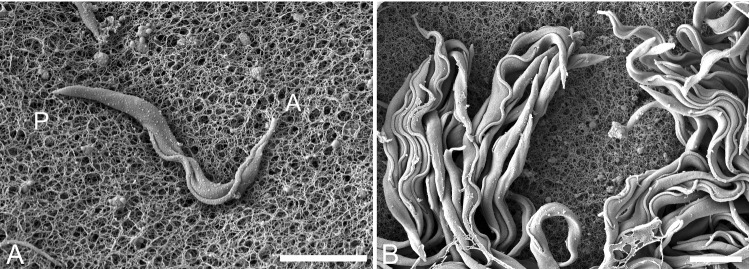
Scanning electron micrographs of single and collective trypanosomes, fixed while swimming on the surface of an agarose gel. **a** A single trypanosome lying horizontally on the gel surface. The diameter of the anterior flagellum tip is smaller than the larger holes of the meshwork. The cell body is wider than the diameter of the largest pores. (A: anterior, free flagella tip, P: posterior). **b** The edge of a dense cell layer exhibiting considerable lateral alignment of cell bodies and flagella. Scale bars: $5\,\upmu \hbox {m}$

The electron micrographs show the size relations of the porous surface mesh with spaces in the range of tens to hundreds of nanometres and the trypanosomes with a width of $2-3\,\upmu \hbox {m}$. The thinnest part of the trypanosomes is the anterior tip of the flagellum, with a diameter of around 250 nm which would allow the insertion of the foremost part of the cell into the mesh, but no significant penetration of the gel by the whole cell body (Fig. 3a). Dense layers of tightly packed cells could be fixed in part, showing close associations between cells, with several groups of cells even showing lateral synchrony in their flagellar wave form (Fig. 3b). Note that the cells were crosslinked by the fixation procedure, but they were not crosslinked with the agarose surface. This corresponds to the observation of a densely packed cell layer in live cell imaging, showing that cells are close enough to bind each other’s surfaces, but, *in vivo*, are free to swim in a narrow fluid surface layer, gliding past each other.

Note, though, that it was not possible to fix the native state of cells as shown in Fig. 2, despite maximal care taken, as the fixation procedure for electron microscopy requires fluid immersion, which inevitably influences the fluid state of the cell layer. Specifically, layers like that in Fig. 3b were perturbed, resulting in movements in and of the cell layer before crosslinking. This means that especially cells at the borders of the cell layer were reordered and partly diluted, resulting in an altered representation of live cell organisation.

Having gained considerable insight into the motile and topological organisation of the trypanosome–gel surface system, we could assess the feasibility of tracking analysis of single-cell behaviour in the migrating fingers, using high spatiotemporal live microscopy.

In order to reproducibly conduct experiments by high resolution, fluorescent live imaging, we downscaled the assay dimensions. The motility phenotypes were reproduced in smaller dishes (diameters 60 mm and 35 mm), which are suitable for conventional fluorescence live imaging systems. The volume of agarose was reduced to 4 ml and 2 ml, respectively, and the volume of the trypanosome drop was reduced to $2\text {-}3\,\upmu \hbox {l}$. The protocol for gel production was optimised for higher reproducibility. This included melting and quenching at constant rates, monitoring gelling and drying conditions in order to produce gels and their surfaces as homogenous, plane and disturbance-free as possible (Methods). Furthermore, we made an initial step towards simplifying the culture medium for cell growth and gel production, in order to eventually gain more insight into the actual mechanism of collective motility in this system. In the fly, the developmental stage of the parasite used here metabolises amino acids, primarily proline (Introduction). This differs from the bloodstream form that utilises glucose, which is nevertheless added to the standard culture media for procyclic culture forms, because of growth advantages for monomorphic variants preferentially used in molecular biology. We omitted glucose as well as hemin and glycerol from all media, which had no significant impact on cell growth (Material and Methods), as had previously been shown for procyclic cells [22].

**Fig. 4:**
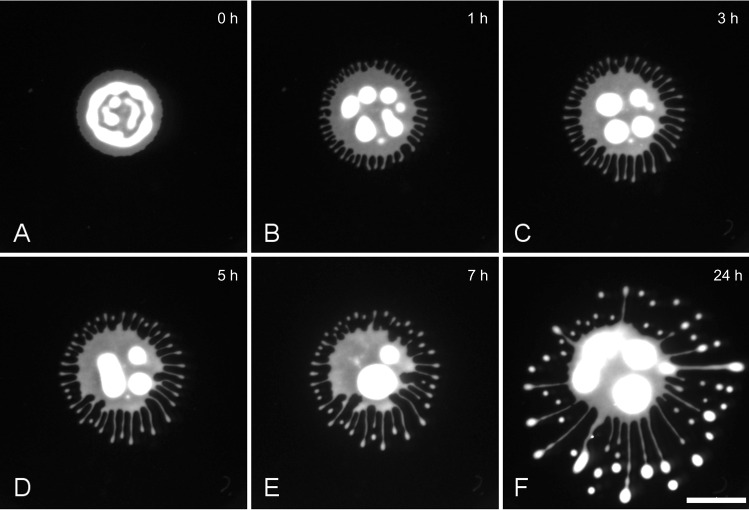
Motility assay on 35-mm agarose gels with $3\,\upmu \hbox {l}$ ($3^{*}10^{6}$ cells) AnTat1.1 dtTomato cells. Compared to the large-scale assay in glucose containing media (Fig. 1), prominent projections formed earlier (**b**) were slightly more numerous and thinner (Fig. S3). **d** 5 h post-inoculation first drops budded off the projection tips, some of which migrated further outward, while the stream of cells left behind retracted or rolled off further **f**. Scale bar: 5 mm

The spreading behaviour of the trypanosome drop was very similar to that in glucose containing assays (Fig. 4). Despite several variations and differences in overall migration patterns, which will be subject of future investigations, the migration speed of projection tips was in a similar range of tens to several hundred $\upmu \hbox {m}$ per hour, fluctuating as described above (Fig. S2). Interestingly the tips of the projections tended to round up and pinch off drops at later time points, more pronounced so in the smaller dishes (Fig. 4e). Importantly, despite all variance, the migration behaviour of projection tips and the general pattern of cell motility was remarkably robust and amenable to tracking analysis.

In order to analyse the swimming behaviour of single trypanosomes in the dense cell mass of projections, we added ${\sim }\,0,3 {\%}$ tdTomato cells to a culture of wild-type cells. Both cell populations were grown to late log-phase (${\sim }\,1^{*}10^{7}$ cells/ml). The cells were centrifuged and resuspended to yield a concentration of ${\sim }\,1^{*}10^{9}$ cells/ml. Three microlitres (${\sim }\,3^{*}10^{6}$ of these cells) was pipetted onto the surface of a 0,4 % agarose gel in a 35-mm- or 60-mm-diameter Petri dish. The dishes were sealed with Parafilm immediately and kept on an automated microscope stage under environmental control at $27\,^{\circ }\hbox {C}$, $5 \,\%\,\hbox {CO}_{2}$. Replicate dishes were spotted with cell drops and incubated in parallel in a cell culture incubator under the same conditions. Expansion of the cell colony on these gels, was recorded regularly using a gel documentation fluorescence reader (Fig. 4). The 60-mm gels allowed migration analysis for longer periods, as migration continued in stable projections for up to 72 h.

**Fig. 5:**
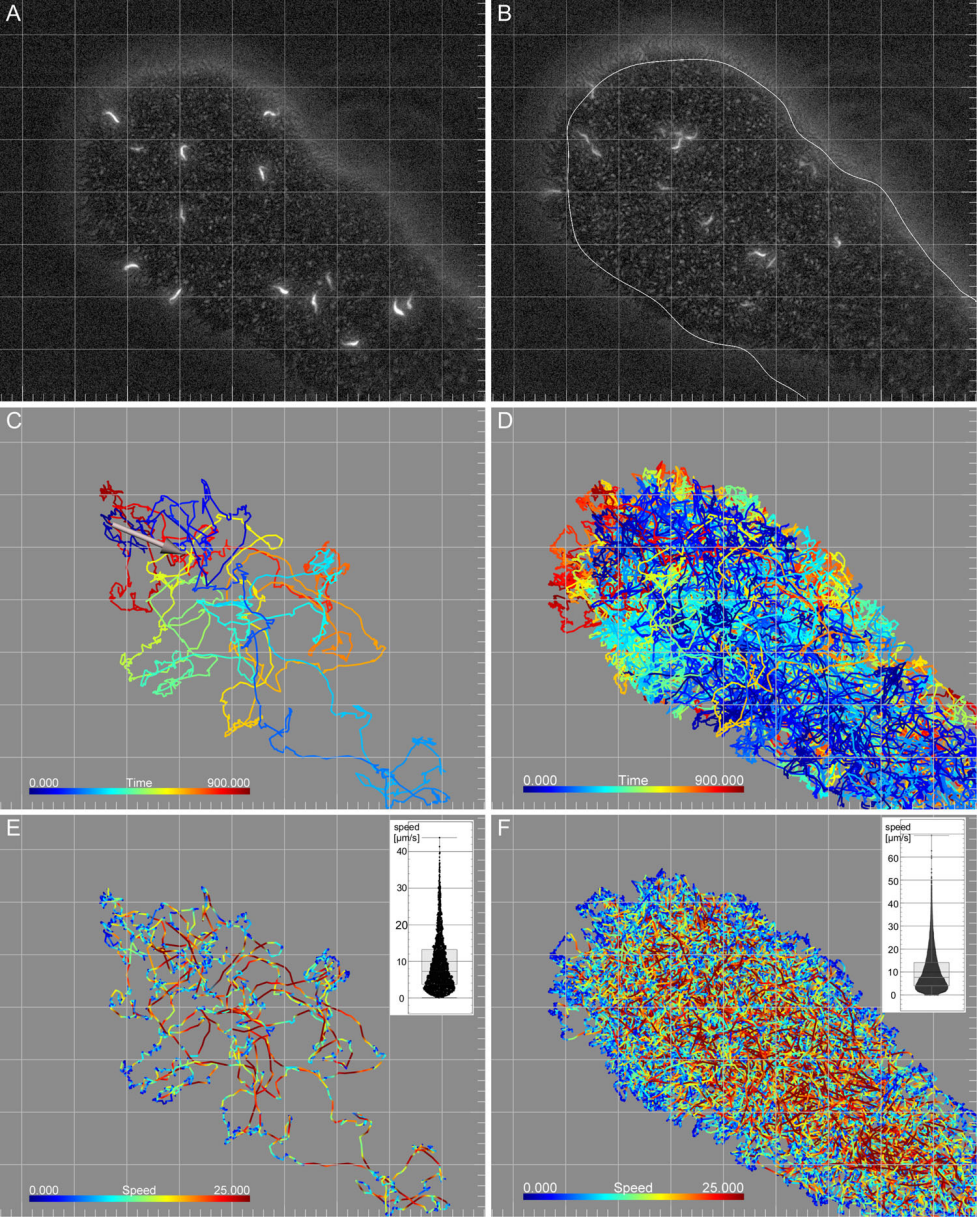
Representative projection tip with tracked fluorescent trypanosomes (0,3 %). **a** First frame of a recorded video in combined fluorescence and phase contrast mode. **b** Last frame of recording at time point 15 min. The line represents the manually traced rim of the projection at time point 0 min in (**a**). Migration speed was $1,4\,\upmu \hbox {m}/\hbox { min }$). **c** Track of a single trypanosome crossing the entire projection area for the length of the recording (15 min). Time is colour-coded and shows the movement of the cell from the tip of the projection to the base at the edge of the image and back again. The arrow marks the net displacement. **d** Tracks of all cells in the area. Time is colour-coded, showing the migration of single cells with the projection rim as seen in (**b**) (red tracks represent later time points in the top left). **e** The single track in **c** shown to be colour-coded for speed (shown in Video S2). Multiple visits to the projection border during which directed swimming motion is halted, are thus visualised in blue. Directional stretches of swimming at speeds above $25\,\upmu \hbox {m/s}$ are visible as red tracks throughout the interior. The inset shows the box plot for the median of the cell’s speed ($7,27\,\upmu \hbox {m/ s}$), plus the speed distribution of all time points of the track. **f** All tracks are shown as in (**d**) colour-coded for speed as in **e**. The periphery is reached by all cells, and their tracks demarcate the border in low-speed blue due to their temporary halts. The interior is uniformly traversed by all cells with stretches of directional swimming and intermediate phases of tumbling and direction changes as for the example cell in **e**. The inset shows the box plot for the median of all cell speeds ($7,68\,\upmu \hbox {m/s}$), plus the speed distribution of all cells and time points. Grid scale: $100\,\upmu \hbox {m}$, Tick mark spacing: $20\,\upmu \hbox {m}$

During this time period, single projection tips were chosen for video microscopy. Recordings were done using simultaneous fluorescence and transmission illumination, in order to track movement of single cells while recording projection border migration. The progression of the distal tip border tangent was measured along the longitudinal axis of the projection (as in Fig. 2C). The projections consisted of a dense single layer of cells, in which the fluorescent cells could be identified in the focal plane at all times (Fig. 5a, b).

The cells were so tightly packed that the discrimination of single cells was not possible in phase contrast. At the projection border, all cells were oriented with their flagellar tip directed outward. The cells showed a tendency towards a parallel alignment, depending on density. Due to this arrangement, single cells were discernible and could be seen to be independently motile, but remained at the rim for timespans up to seconds. Fluorescence imaging allowed the motility analysis of single cells in the entire area, showing constant active motility of all cells in the interior regions as well as intermediate stops of varying length at the border, after which the cells returned to the interior region, either by reversing flagellar movement, or by turning and continued forward swimming.

The cells were recorded at 4 fps for 15 min. During this time, the distal projection front progressed at speeds between 0 (no progression) and $4\,\upmu \hbox {m}/\hbox { min}$. Each field of view was chosen to contain a projection tip $250\text {-}400\,\upmu \hbox {m}$ in length and $200\text {-}300\,\upmu \hbox {m}$ in width. In the timespan of the recording, the projection contained between 10–40 fluorescent cells, which were tracked using Imaris. At this temporal resolution (3600 frames/15 min), tracking resulted in roughly 40,000–100,000 instantaneous speed measurements per recording. The single tracks exhibit speed values ranging between 0 and up to ${\sim }\,80\,\upmu \hbox {m/s}$ with a median speed between 3 and $8\,\upmu \hbox {m/s}$ (Fig. 5e, f). The tracks show fast directional swimming of each cell, interrupted by slower phases including direction changes and residence times at the fluid border.

The cells did not show a swimming direction bias in the migrating projection tips (Fig. 5c, e, Video S2 and Fig. S4). Except for the outward orientation and alignment at the projection border, every cell was unrestricted in the direction of movement. Despite the apparent local freedom of movement, the majority of fluorescent cells stayed in the observed area for most of the recording period. One typical example track shows a cell navigating the area of the projection tip for the duration of the recording (Fig. 5c, e and Video S2). On average, there was no net influx of fluorescent cells. The turnover rate in the observation period was in the range of the number of tracked cells per time point. For example, in the recording shown in Fig. 5, on average two cells left the field of view, while two entered every minute. The behaviour of single cells was very similar to those in the original assay, in which we had attempted the tracking experiment using a fluorescence stereo microscope (Video S3).

**Fig. 6:**
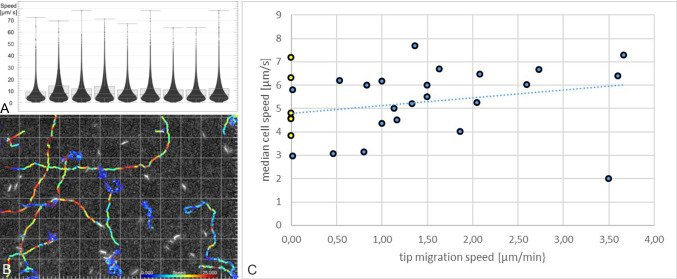
Median cell speeds in projection tips show no significant correlation with projection migration speed. **a** Instantaneous cell speeds measured by tracking of fluorescent cells in single, migrating projection tips, as shown in the insets in Fig. 5e, f. Data from various projections in one experiment with $25{\mathrm{th}}$ and $75{\mathrm{th}}$ percentiles of median speed distribution. Pearson’s correlation coefficient (r) $=$ 0,12. **b** Representative tracks of single trypanosomes in controls, recorded in concentrated drops without gel support. Colour-coded for instantaneous speed. **c** Data from different experiments in blue. Each data point represents the median of all instantaneous cell speeds measured in one projection tip, r $=$ 0,23. Tracked data were recorded from various projections and time points during a period of 72 h. Median speeds of controls are shown in yellow

To evaluate a possible correlation of bulk cell motility with the speed of collective migration, the median of all instantaneous speed values in each projection was plotted against the migration speed of the projection tip (Fig. 6c). The control data are shown in yellow at zero migration speed. Figure 6a shows the results from nine different projections and time points of one experiment, including the data from Fig. 5.

As a control, the concentrated cells were also tracked in a humid chamber, pipetted directly into a plastic Petri dish and covered with a 10-mm-diameter cover slip. The packing rate was lower than observed on gels, but the cells were concentrated as much as possible in a thin fluid layer (${\sim }\,10\,\upmu \hbox {m}$ height), without underlying semi-solid support and without impeding viability. The cells in the centre region of the cover slip could be reliably tracked in the focus plane for at least 1 h under these conditions with constant motile behaviour. The swimming trajectories of most cells were more directional than those in projections on gel surfaces, reflecting missing lateral confinement in the plane beneath the coverslip (Fig. 6b). Despite these differences, the motility behaviour of non-border cells was equivalent and the median values of instantaneous cell speeds were in a similar range of $3-8 \upmu \hbox {m/s}$ (Fig. 6c).

There was no significant correlation between the average of single-cell speeds and projection migration speed. The spatiotemporal resolution of our data was more than sufficient to enable the detection of regional variations of collective motility patterns, but none such were found (*e.g*. accelerated motility before entry into border regions, or directional alignment of cells in the direction of migration, *etc*.). In other words, although a more thorough investigation of the acquired data remains to be undertaken, so far we found no evidence of specific collective motility behaviour directly causing the directional movement of the projection borders. The data remain to be combined with results from experiments analysing aspects of the gel/trypanosome system other than cell motility, *e.g.* fluidity aspects of the gel surface.

## Discussion

We describe here the customisation of a commonly used collective motility assay in trypanosome research, facilitating qualitative and quantitative analysis of single-cell behaviour of a flagellate microswimmer in crowded environments and confinement. We have downscaled an agarose gel system which allows the radial expansion of dense groups of millions of cells in characteristic finger like projections similar to the collective swarming behaviour of some bacteria. Importantly, the cellular basis of the trypanosome social motility assay has not been elucidated to date. It is unknown how the cells interact in colonies or if the apparent swarming behaviour is a consequence of a change in motile behaviour elicited by cellular interactions. Neither are the physicochemical grounds for social motility known, as specific excreted products, known to drive swarming in prokaryotes, have not been identified yet. Therefore, we also imagine this work to be helpful in determining the actual mechanism of social motility in trypanosomes.

Our analyses in this work show the arrangement of dense masses of procyclic culture forms, swimming in a micrometre thin, single layer between the surface of a hydrogel mesh containing nutrient medium and an air interface. The single fluorescent cells that were tracked in this conglomerate, while it was directionally migrating, were shown to swim with typical speeds during persistent forward motion, freely in any direction. The very weak correlation of projection migration speed and average cell speed, as well as the lack of directional bias, led us to conclude that directional motility of groups of cells, also known as swarming, was not responsible for the expansion of the trypanosome layer on the agarose gel surface.

The confinement in the projection layer led to a direct halt of forward movement at the projection border, presumably both because the cells swam into the sub-micrometre narrow space of the flat angled fluid–gel–air interface, and due to the insertion of the flagellar tip (250 nm) into holes of the agarose gel mesh (~300 nm). This halt was temporary, as the cells did reverse and swim back into the interior regions of the projection. Interestingly, most cells were aligned laterally in the border region and during their residence time there, forced newly arriving cells to squeeze in between. This palisade-like arrangement is reminiscent of alignments in the fly tissue and could be characteristic for a beginning higher packing order and synchronisation events at high confinement levels. The outward-facing flagellar tips can produce significant fluid flows into the interior of the colony and could thus cause a steady influx of diffusing substances.

Only rarely was a group of aligned trypanosomes seen to actively push forward and thus directly move the projection border. Cells were usually free to slide forwards and backwards between two neighbouring cells, and the entire front could also move laterally along the projection border without causing directed movement of the rim. This means that despite considerable crowding and confinement, the combined cell and gel surface layer supplied enough fluidity for the cell layer to stay dynamic. The question remains: What actually produces the force to slowly but steadily move the projection along the surface? Is it the combined motile force of cells producing pressure normal to the fluid interface and if so, why is the migration mainly radial along the long axis of the projection? Is it the undirected movement in the interior that produces a pressure field that acts on the dynamically stable borders? Are short-range interactions responsible, or do long-range effects come into play, potentially the dynamic state of the entire colony? Or are there significant fluid forces that are produced by, but not necessarily reflected in, motility behaviour of the single cells? Are there dominant external fluid forces, *i.e*. from the surrounding naive gel surface, or from the interior of the gel?

The last question needs to be addressed by changing the gel surface and fluid composition of the system. We have begun to simplify the media used to culture the cells and produce the gels, in the hope of eventually making their physicochemical properties more controllable. Reduction of as many factors as possible should be attempted, in order to perform addback experiments to identify the contribution of specific reagents. This also includes the availability of nutritional components and the metabolic changes the trypanosomes are capable of, which probably also influence group behaviour. Specifically, a first further step will be the separation of motility and cell growth, to discern what is the dominant driving force of radial expansion in the first place. Is the colony simply expanded by growth, or is motility, combined with fluid dynamics, sufficient to produce collective motion?

The questions regarding the active role of the trypanosomes are analysable by changing the type of cells and manipulating these. The capability to do this is a huge advantage of the trypanosome system. Several natural morphotypes are available and genetic manipulation readily produces specific variations. The social motility assay used so far has used a simple readout: motile or non-motile on agarose surfaces. With the direct observation of cell motility as described here, the assay can be used to its full potential. When the forces acting to produce the fascinating patterns of group motility are controllable, trypanosome factors will be found that specifically influence motile behaviour in well-characterised, confined fluid spaces. This could give us valuable insight into the behaviour of the parasite in the fly, but also provide a unique microswimmer system to study motility and dynamics of a rich, living active fluid system.

## Supplementary Information

Below is the link to the electronic supplementary material.

Supplementary material 1 (tif 2342 KB)

Supplementary material 2 (tif 1558 KB)

Supplementary material 3 (tif 297 KB)

Supplementary material 4 (tif 1428 KB)

Supplementary material 5 (mp4 9717 KB)

Supplementary material 6 (mp4 9188 KB)

Supplementary material 7 (mp4 10134 KB)
