# Supplementary figures and images for: Single-cell motile behaviour of Trypanosoma brucei in thin-layered fluid collectives
Source: Eur Phys J E Soft Matter. 2021 Mar 23;44(3):37. doi: 10.1140/epje/s10189-021-00052-7 (PMC7987620; doi:10.1140/epje/s10189-021-00052-7)

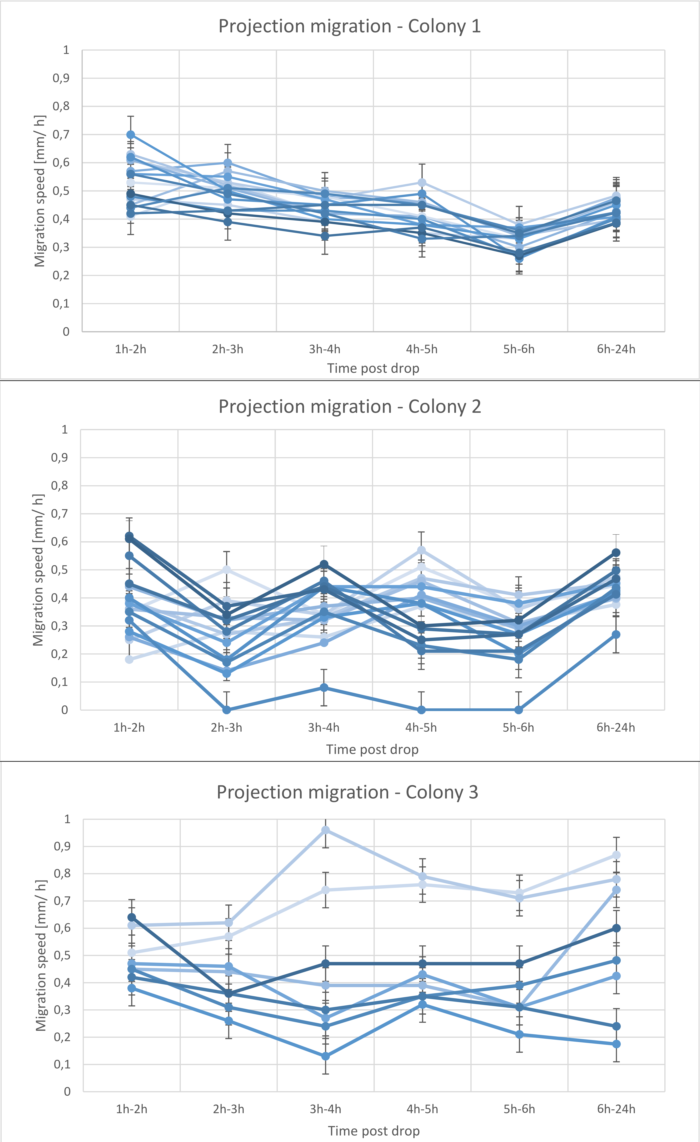

Supplement: Supplementary file 1 — Supplementary material 1 (tif 2342 KB) [file 10189_2021_52_MOESM1_ESM.tif]

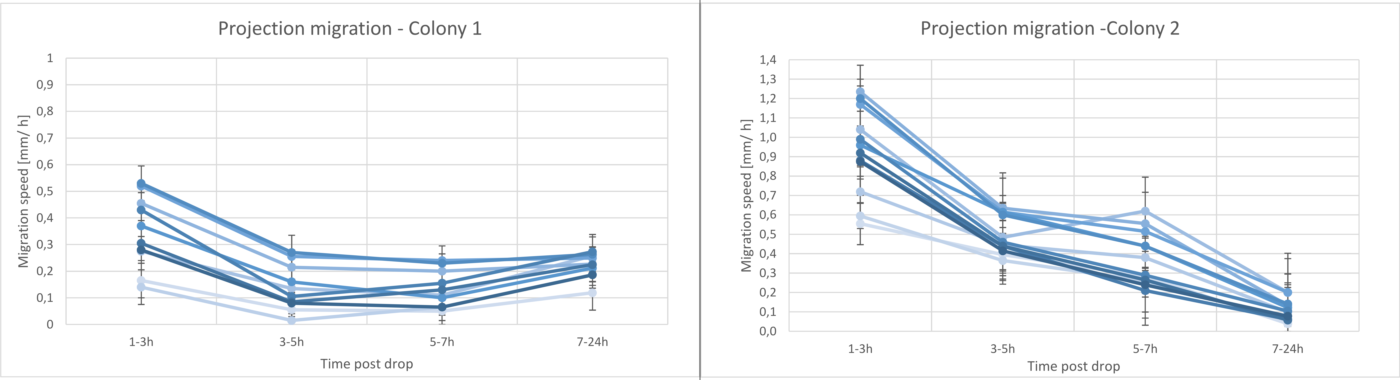

Supplement: Supplementary file 2 — Supplementary material 2 (tif 1558 KB) [file 10189_2021_52_MOESM2_ESM.tif]

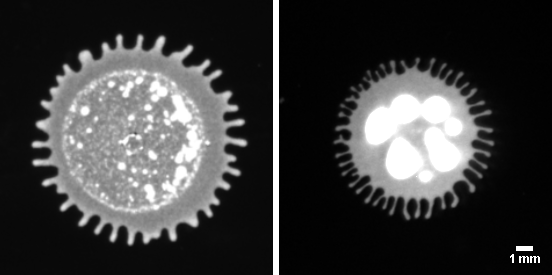

Supplement: Supplementary file 3 — Supplementary material 3 (tif 297 KB) [file 10189_2021_52_MOESM3_ESM.tif]

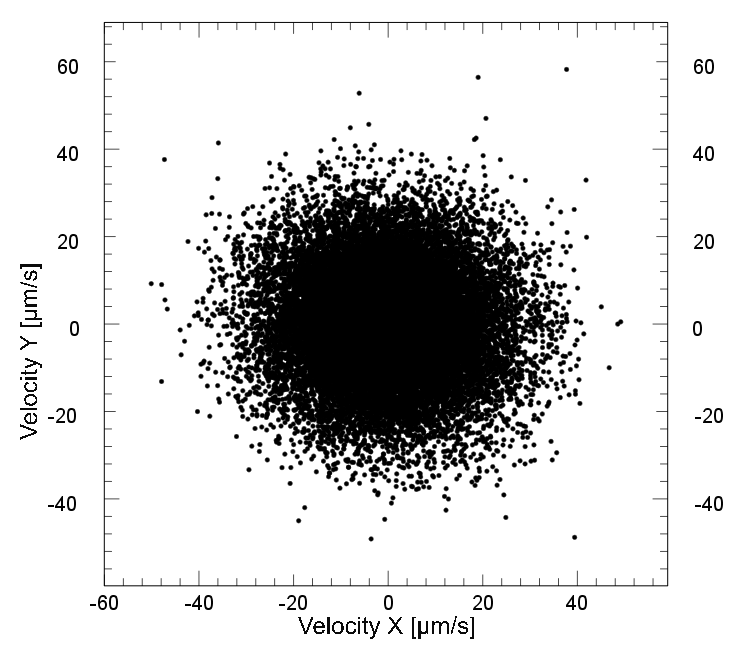

Supplement: Supplementary file 4 — Supplementary material 4 (tif 1428 KB) [file 10189_2021_52_MOESM4_ESM.tif]
